# Supplementary material for: Climate extremes in Svalbard over the last two millennia are linked to atmospheric blocking
Source: Nat Commun. 2024 Jun 3;15:4432. doi: 10.1038/s41467-024-48603-8 (PMC11148056; doi:10.1038/s41467-024-48603-8)
Supplement: Supplementary file 1 — Supplementary Information [file 41467_2024_48603_MOESM1_ESM.pdf]

Supplementary Information for

Climate extremes in Svalbard over the last two millennia are linked to atmospheric blocking

Francois Lapointe[1,2], Ambarish V. Karmalkar[3], Raymond S. Bradley[1,2], Michael J. Retelle[4,5], Feng Wang[6]

[1]Department of Earth, Geographic and Climate Sciences, University of Massachusetts, Amherst, MA, USA.

[2]WCRP Climate and Cryosphere (CliC) Project, University of Massachusetts, Amherst, MA, USA.

[3]Department of Geosciences, University of Rhode Island, Kingston, RI, USA.

[4]Earth and Climate Sciences, Bates College, Lewiston, ME, USA.

[5]Department of Geology, The University Center in Svalbard, Svalbard, Norway.

[6]Institut National de la recherche scientifique, University of Québec, Québec, Canada.

The supplementary materials include:

Supplementary Figures 1 to 22.
Supplementary Table 1.
Supplementary Notes 1 and 2.

Year	Month	Day	Time (UTC+1)	RR_12 [mm]	T [°C]	AC
1959	11	11	19	10.0	4.5	SWc
1966	12	28	7	18.0	3.2	Sc
1983	3	2	7	13.0	3.6	SWc
1984	3	10	19	12.0	2.3	Wc
1986	3	11	19	12.4	2.1	SWc
1991	12	7	19	18.2	4.6	SWc
1991	2	1	19	11.0	3.8	SWc
1993	11	30	19	11.8	4.4	SWc
1994	2	13	7	14.8	0.3	SWc
1995	12	2	19	19.2	2.8	SWc
2008	1	2	19	11.1	1.7	SWc
2009	12	10	19	11.0	4.4	Sa
2011	3	17	19	18.2	3.5	SWc
2012	1	30	7	21.2	4.1	SWa
2012	1	30	19	10.4	3.8	SWa
2016	1	2	19	16.3	2.3	SWc
2016	11	7	19	17.2	5.0	SWc
2016	11	8	7	24.5	2.3	Wc
2017	2	7	19	10.0	3.3	SWa
2018	1	13	19	15.5	4.4	SWc
2018	2	27	19	11.0	1.5	Bc

Table S1. List of days with heavy rainfall events (>10mm) between November and March at Longyearbyen and Svalbard airport since 1958. From Dobler et al. 2019[1]

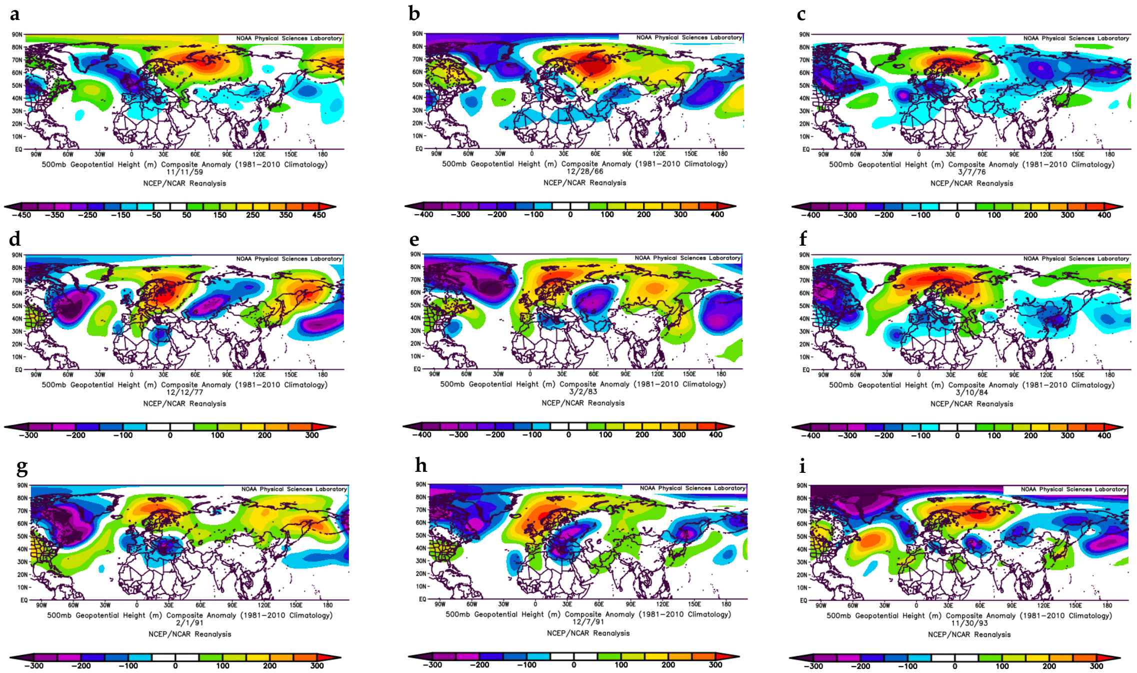

Figure S1. The daily 500hPa (z500hPa) map anomalies (relative to the 1981-2010 climatology) and the amounts of rainfall for all the days with rainfall >10mm as seen in Table S1. **a** z500hPa anomalies for November 11, 1959: 10mm. **b** z500hPa anomalies for December 28, 1966: 18mm. **c** z500hPa anomalies for March 7, 1976 : 13mm. **d** z500hPa anomalies for December 12, 1977: 15mm. **e** z500hPa anomalies for March 2, 1983: 13mm. **f** z500hPa anomalies for March 10, 1984: 14mm. **g**, z500hPa anomalies for February 1, 1991: 18.2mm. **h** z500hPa anomalies for December 7, 1991: 22mm. **i** z500hPa anomalies for November 30, 1993: 16mm.

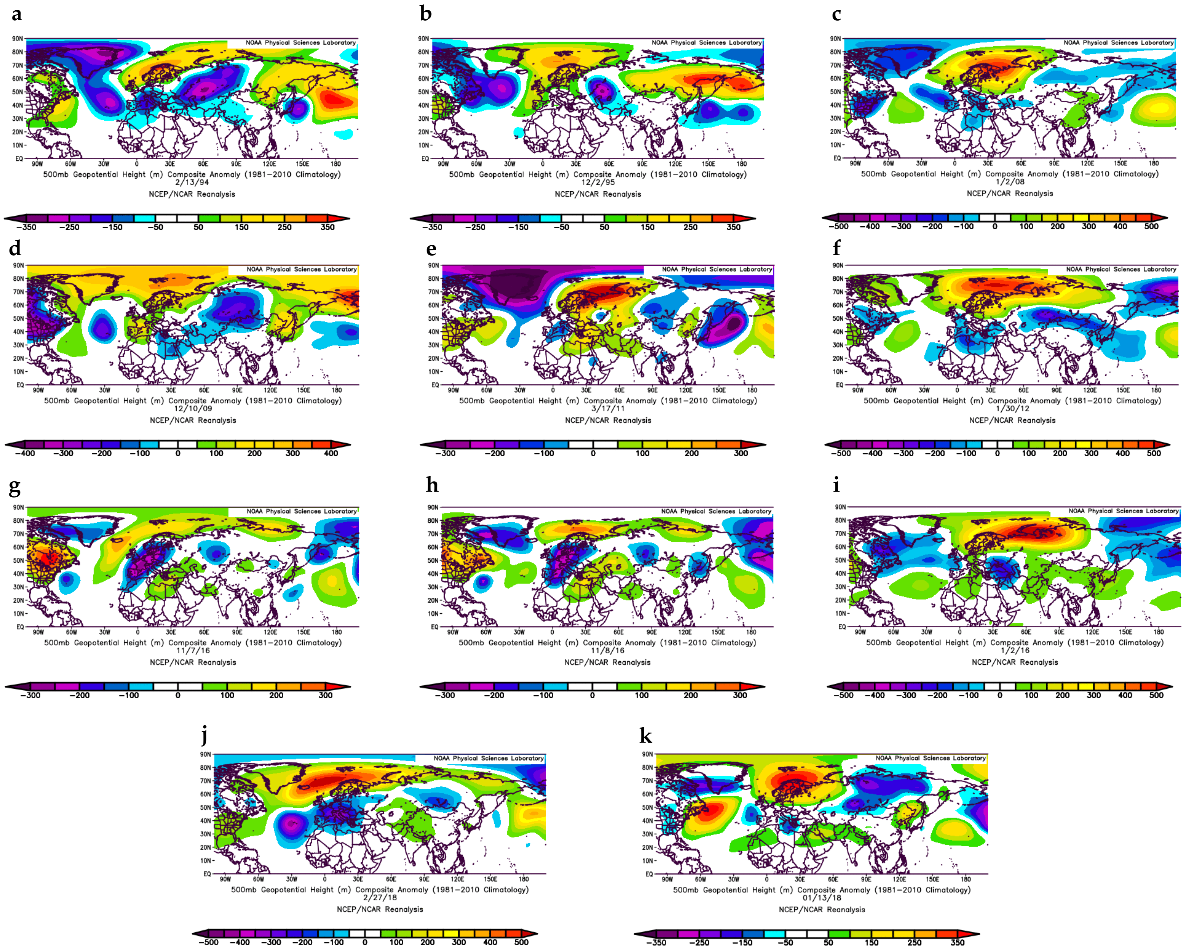

Figure S2. Continuation of Fig. S2. **a** z500hPa anomalies for February 13, 1994 : 14.8mm. **b** z500hPa anomalies for December 2, 1995: 19.2mm. **c** z500hPa anomalies for January 2, 2008: 11.1mm. **d** z500hPa anomalies for December 10, 2009: 11.2mm. **e** z500hPa anomalies for March 17, 2011: 18.2mm. **f** z500hPa anomalies for January 30, 2012: 21.2mm. **g** z500hPa anomalies for November 7, 2016: 19mm. **h** z500hPa anomalies for November 8, 2016: 24.5mm. **i** z500hPa anomalies for January 2, 2016: 16.3mm. **j** z500hPa anomalies for February 27, 2018: 11mm. **k** z500hPa anomalies for January 13, 2018: 15.5mm

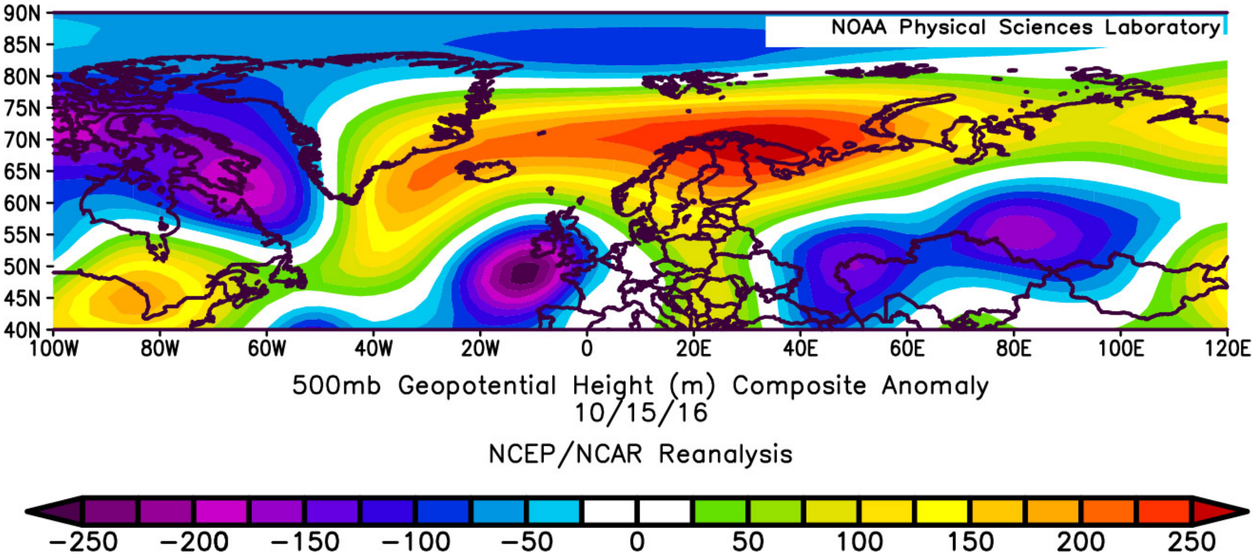

Figure S3. Atmospheric anomaly for October 15, 2016 (relative to the 1981-2010 climatology), a day with unprecedented rainfall amount in Svalbard. Geopotential height data from Kalnay et al.[2].

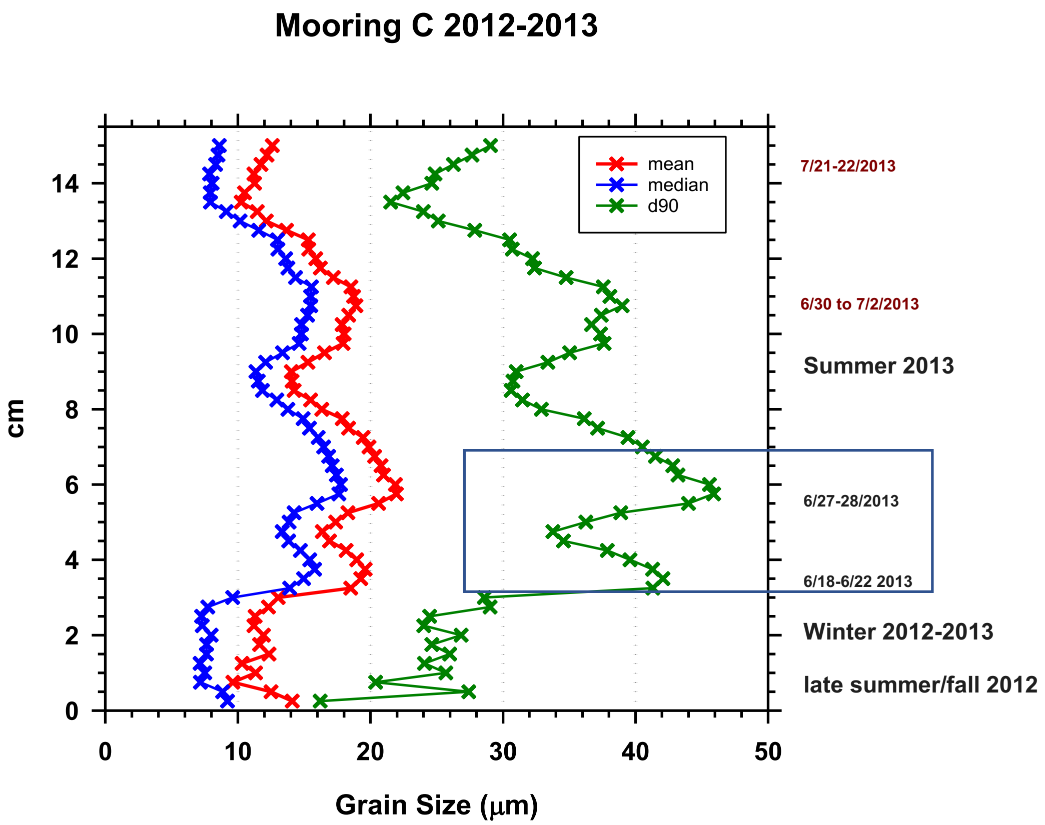

Figure S4. Sediment trap from 2012-2013 showing grain-size variability. See methods and supplementary text 2 for more information.

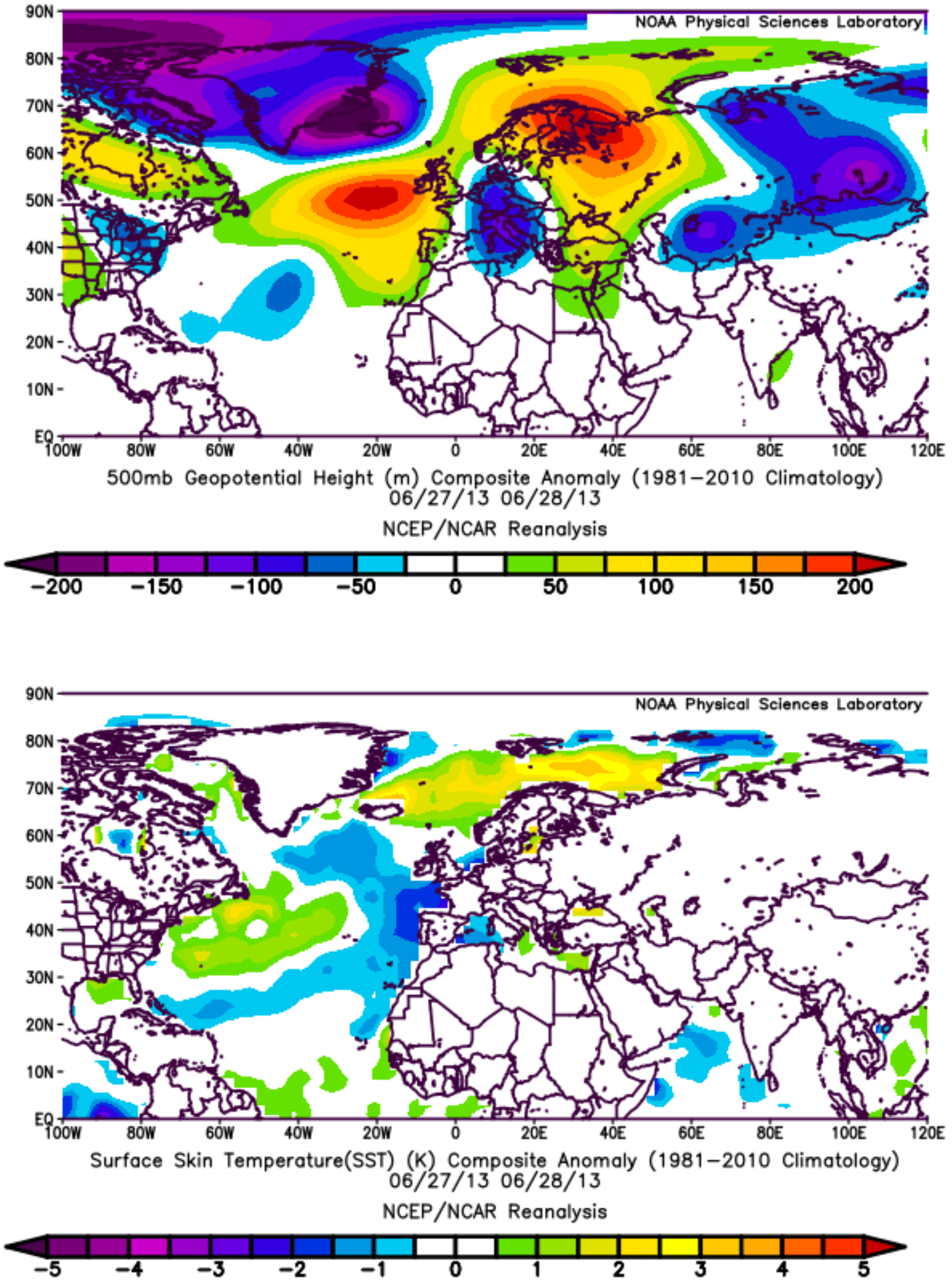

Figure S5. Upper panel: Atmospheric conditions (z500hPa)[2] and lower panel: sea surface temperature[2] on 6/27/2013 to 6/28/2013.

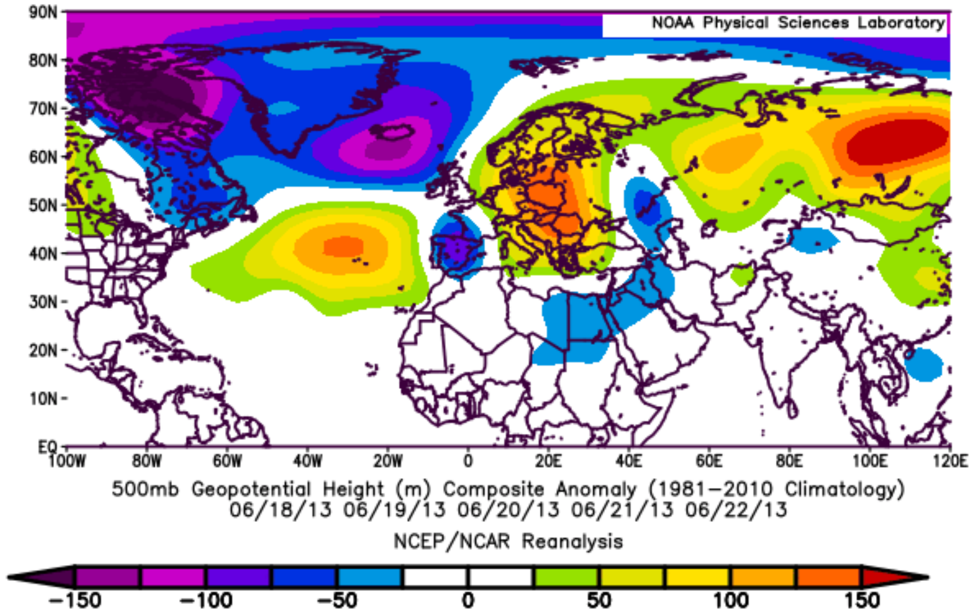

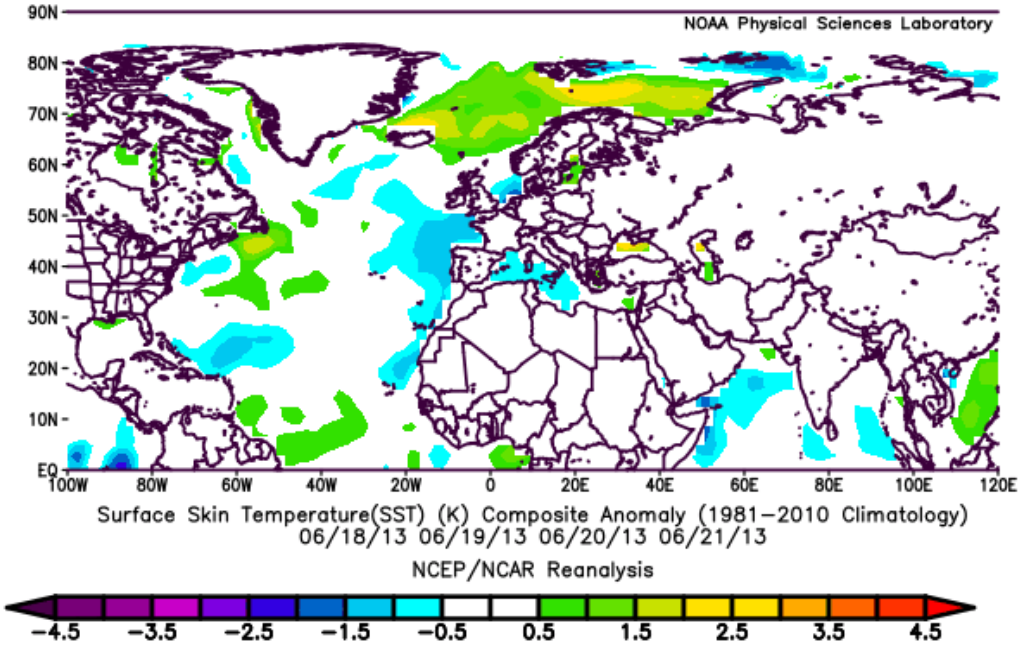

Figure S6. Same as Fig. S12, but on 6/18/2013 to 6/22/2013.

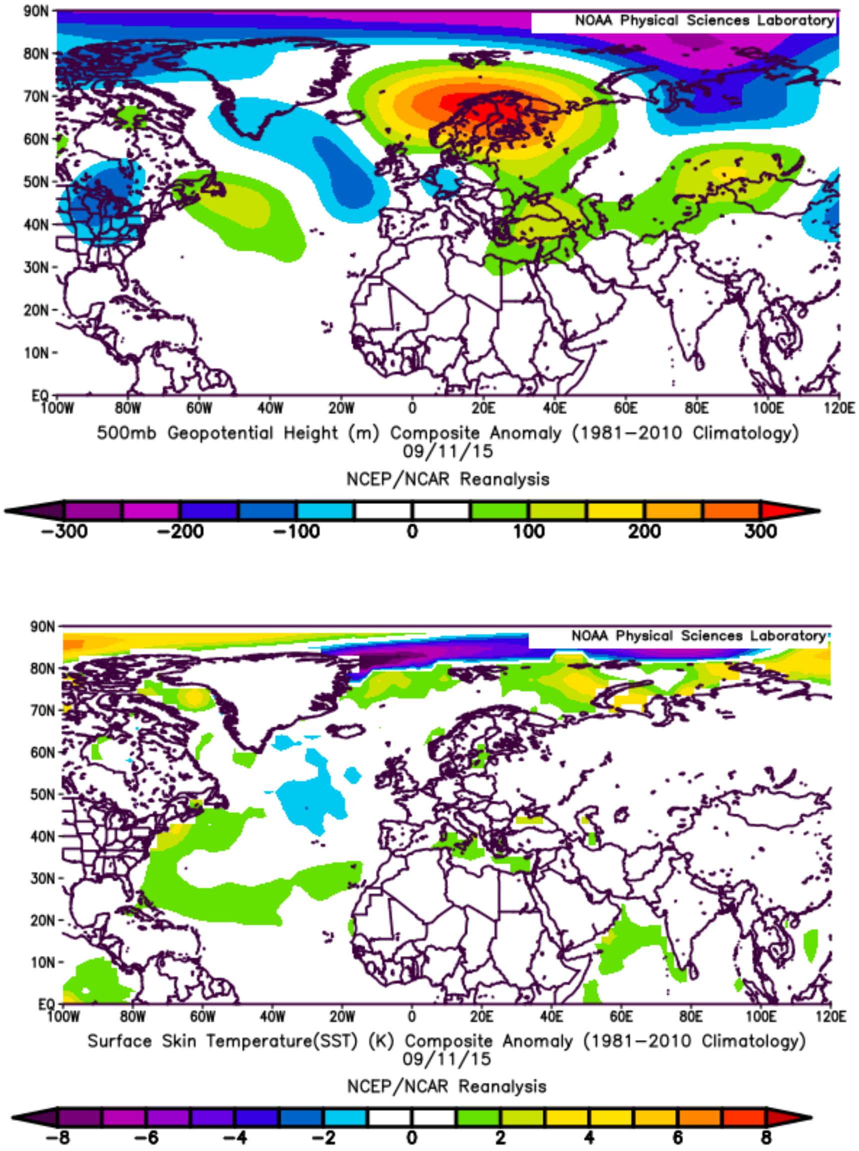

Figure S7. Same as Fig. S12, but on 09/11/2015.

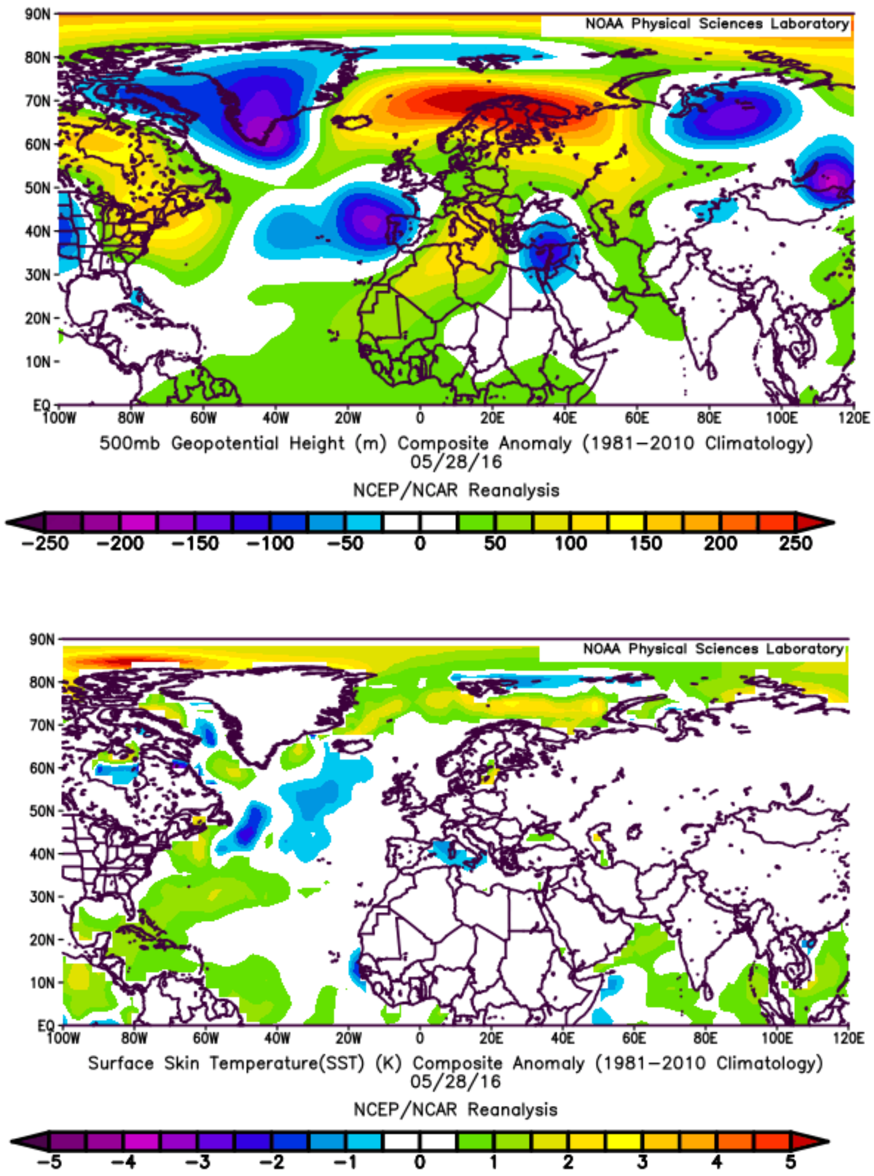

Figure S8. Upper panel: Atmospheric conditions (z500hPa) and lower panel: sea surface temperature on 05/28/16.

Same as above, but for year 2017-2018

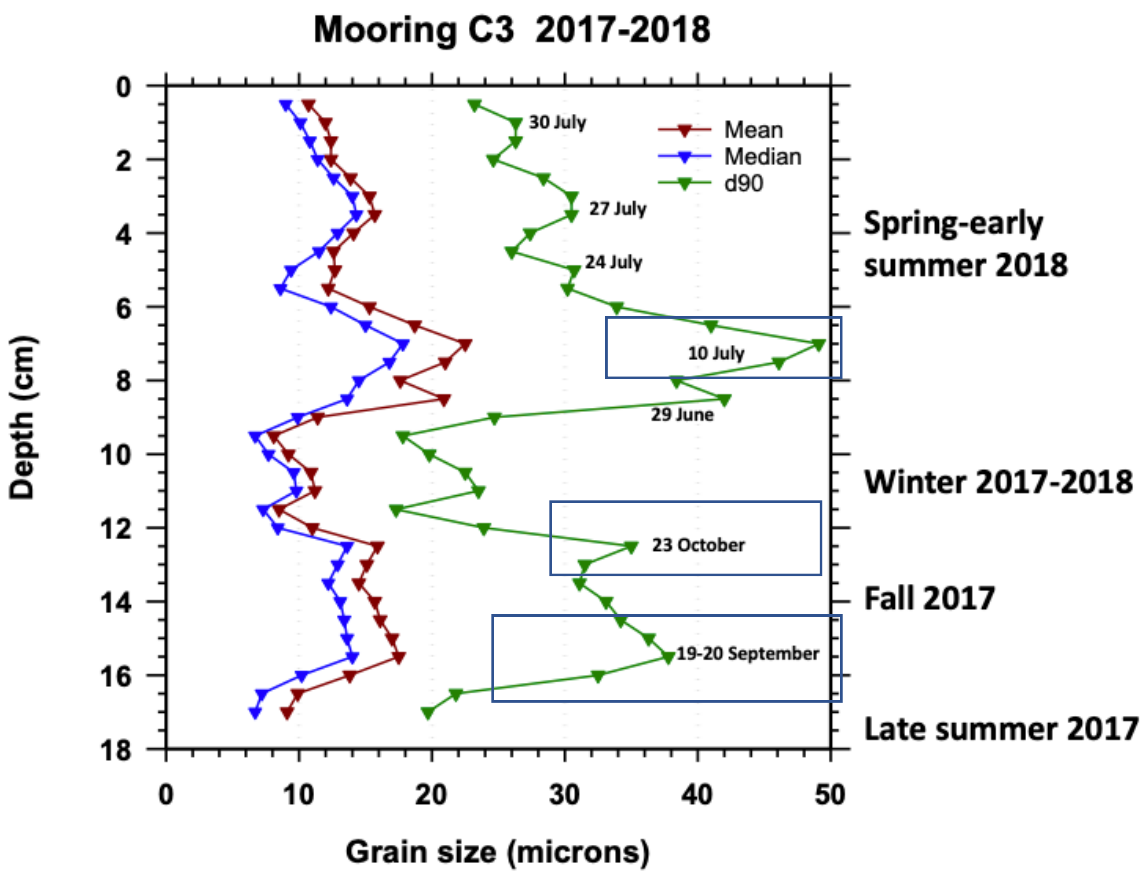

Figure S9. Sediment trap from 2017-2018 showing grain-size variability.

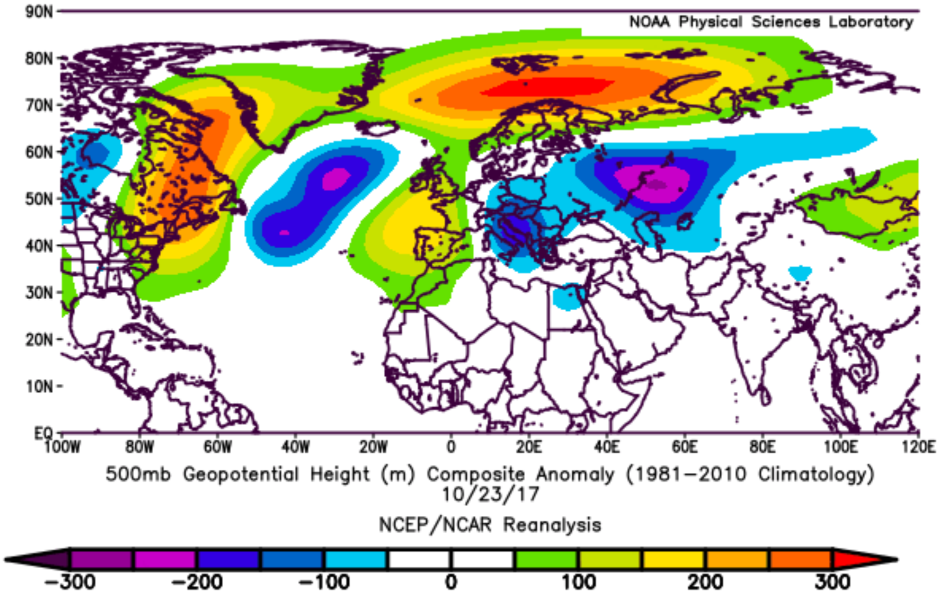

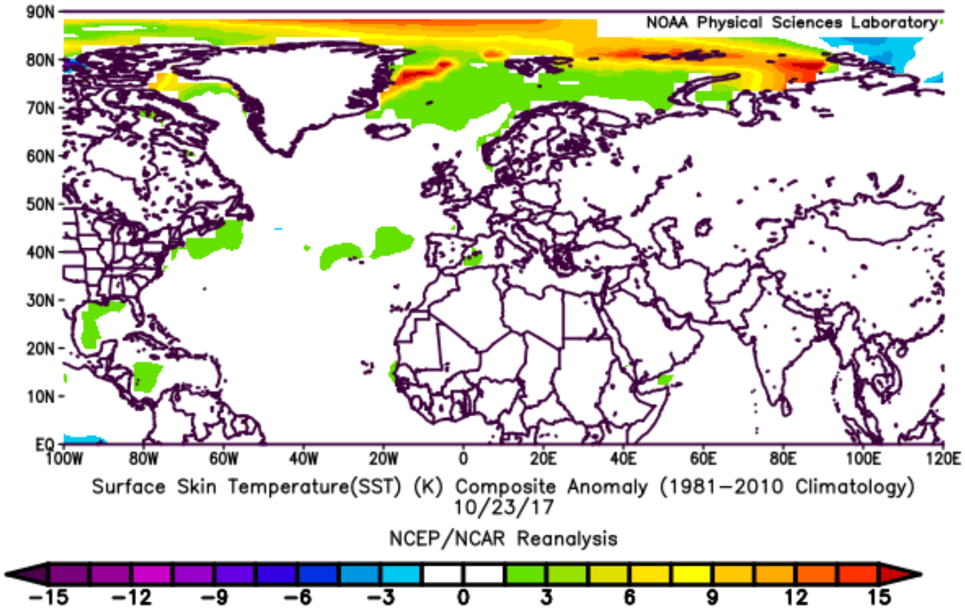

Figure S10. Upper panel: Atmospheric conditions (z500hPa) and lower panel: sea surface temperature on 10/23/2017.

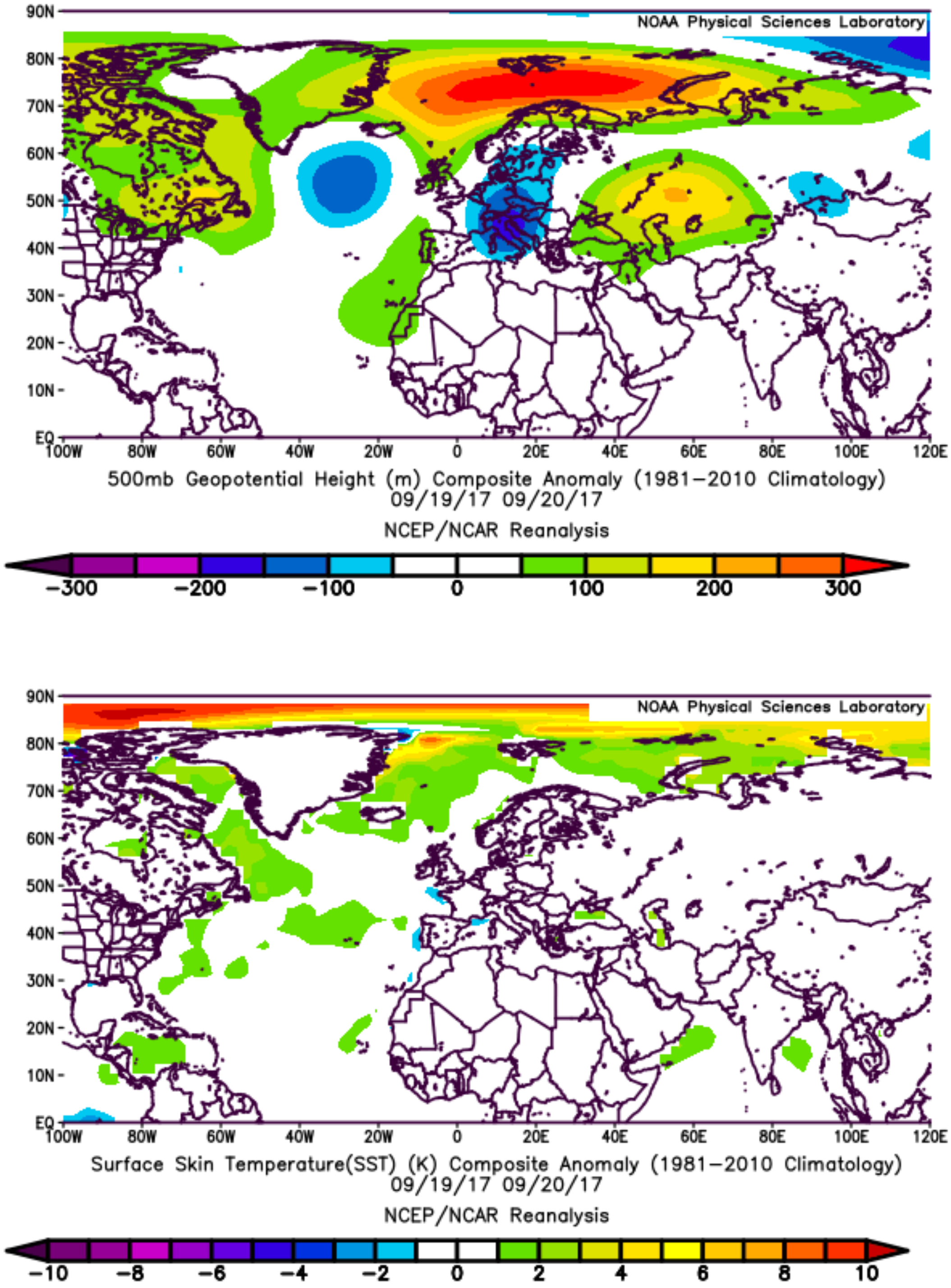

Figure S11. Same as previous, but on 09/19/2017 to 09/20/2017.

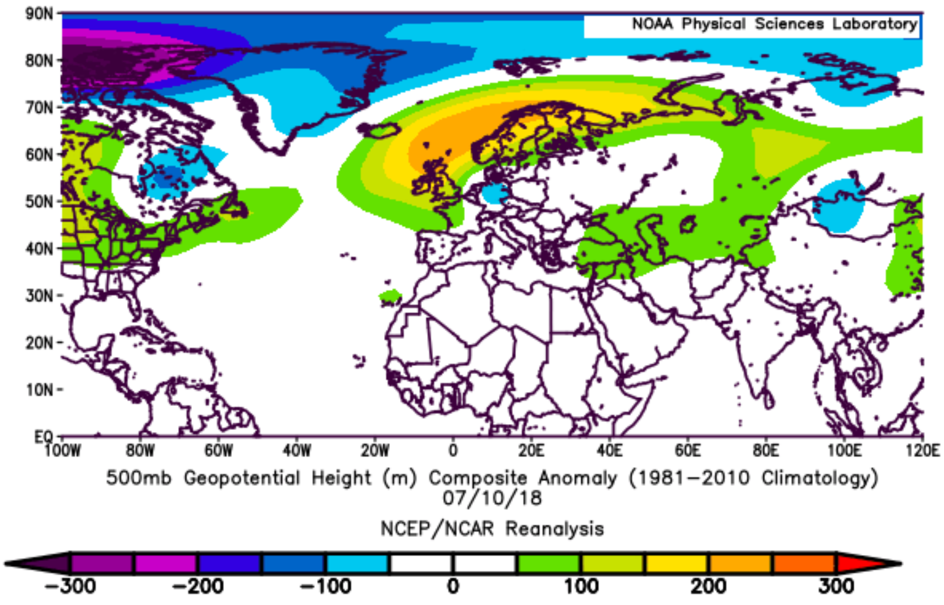

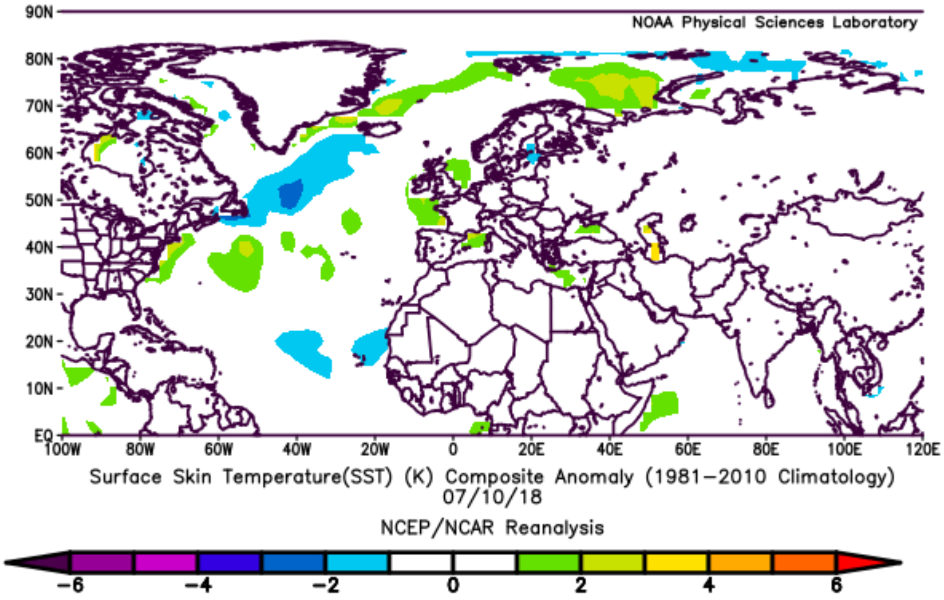

Figure S12. Same as previous but on 07/10/2018.

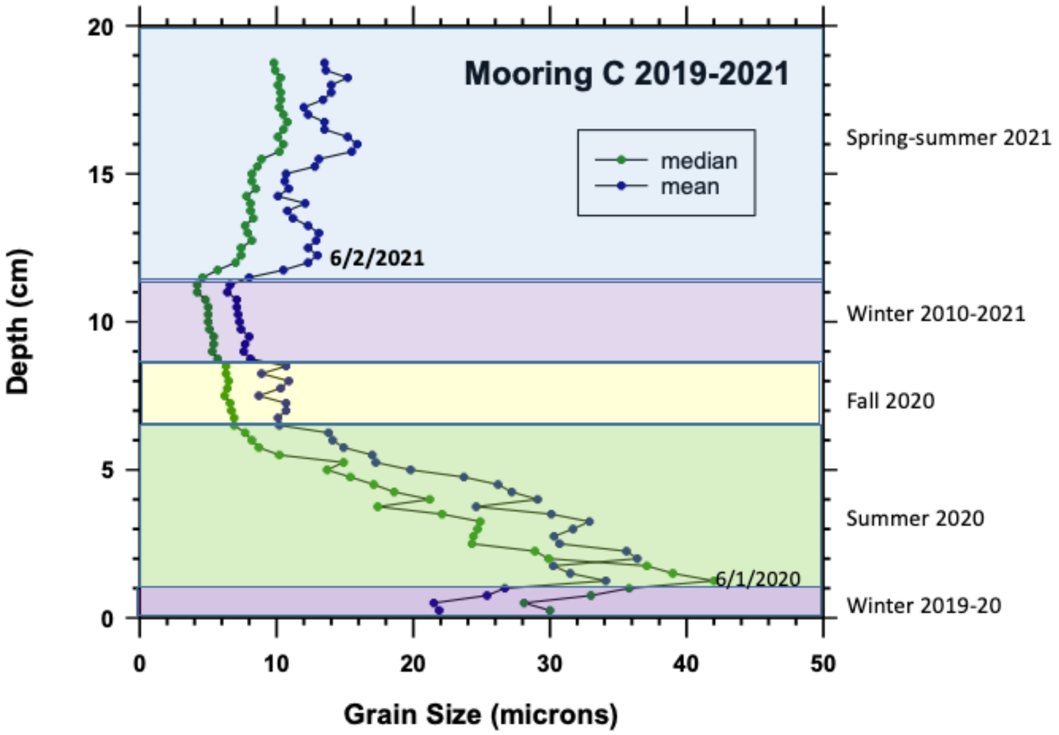

Figure S13. Sediment trap from 2019-2021 showing grain-size variability.

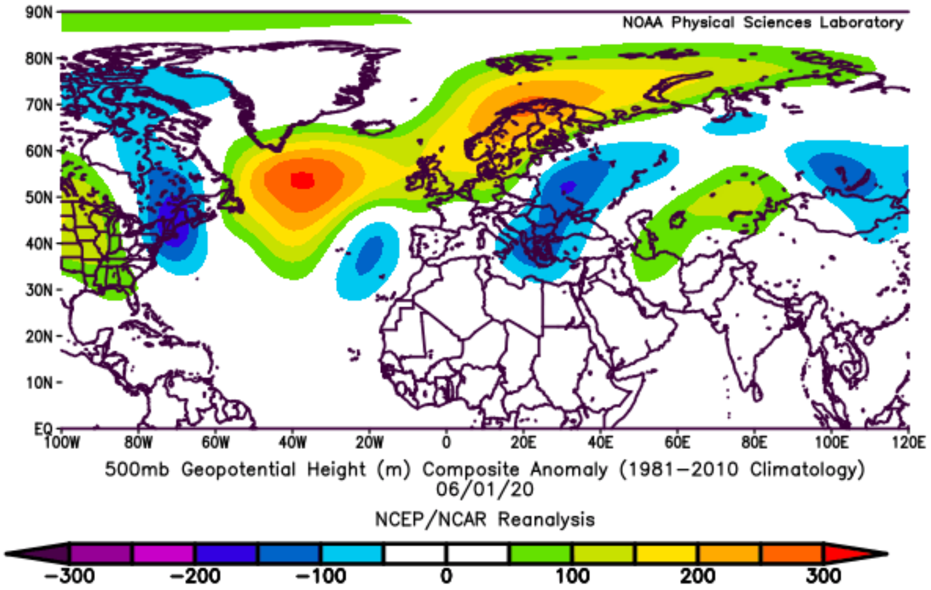

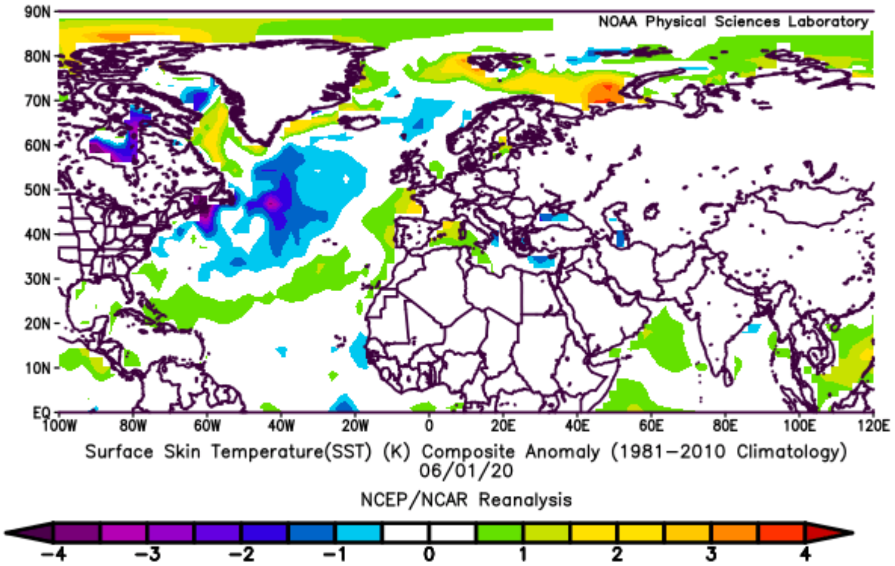

Figure S14. Atmospheric conditions (z500hPa) and sea surface temperature on June 1st, 2020.

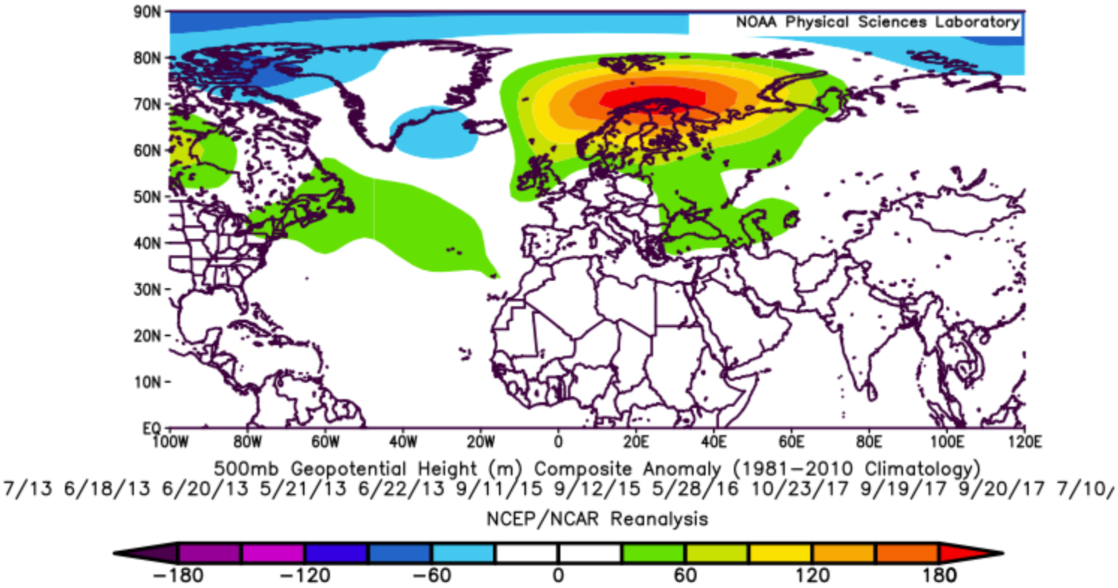

Figure S15. Composite pressure pattern during days with coarsest grain-size in Linnevatnet.

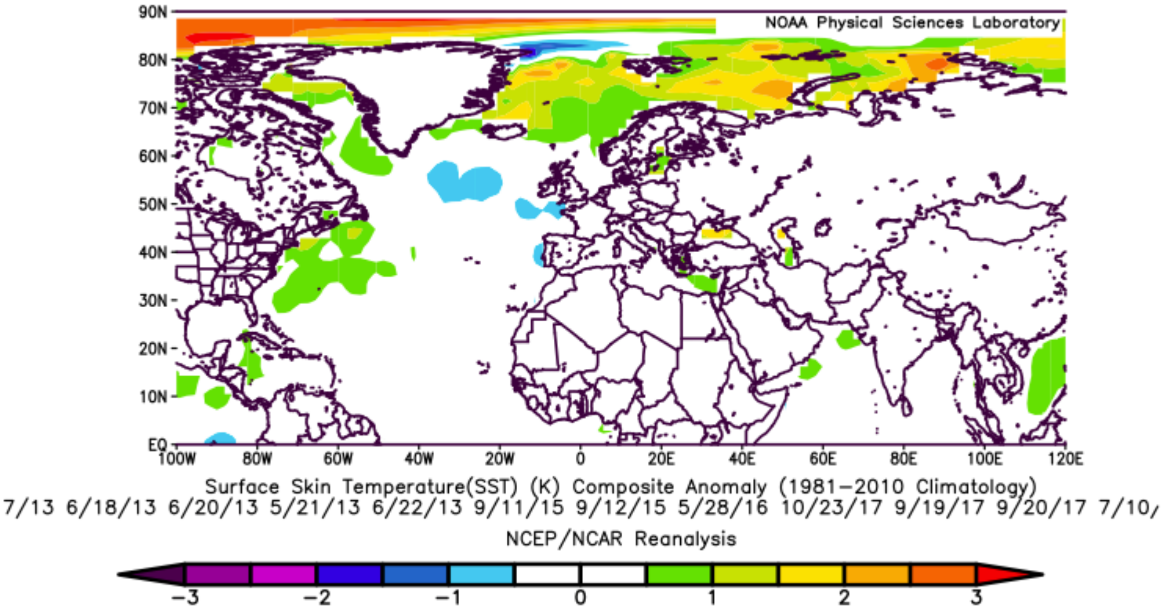

Figure S16. Composite SSTs pattern during days with coarsest grain-size in Linnevatnet.

Supplementary note 1

The recent varve chronology is confirmed by radionuclide ^{137}Cs levels that were detected in a sediment core retrieved from the same coring location (mooring C) in 2016[3]. We cross-referenced sediment layers from thin sections of our core and the core retrieved in 2016 and our varve count matched the ^{137}Cs peak[4]. To validate the most recent chronology, we utilized thin sections from cores acquired in 2005, 2016, and 2018[4].

In order to validate a varve chronology spanning approximately ~1,500 years, we relied on three radiocarbon dates obtained from terrestrial plants at the nearby site 06 (see Figure 1 in Lapointe et al.[4]), which has a sedimentation rate twice that of mooring C[5]. We could not find any macrofossils in our coring locations, so these dates were instrumental in anchoring the chronological framework by employing a chemo-stratigraphic approach (see below). We correlated the calcium carbonate ($CaCO_3$) percentages from Svendsen and Mangerud[6] with μ-XRF calcium variations observed in our study. This correlation allowed us to pinpoint the ^{14}C ages derived from macrofossils at the proximal site and apply them to the composite depth profile derived from our latest core samples. Subsequently, the uncalibrated ^{14}C ages were calibrated using OxCal software, employing the IntCal20 calibration curve[7,8]. The resulting age model was established based on the average varve counts. We acknowledge that there is no chronological constrains for the period ~1-750CE, however, the sedimentation rates do not change significantly until reaching ~370 cm depth when a sudden increase in varve thickness along with carbonate content occurs from 500 cm to 370 cm. The bottom of the sediment composite is characterized by black layers (monosulfidic laminae) indicative of meromixis conditions[4,6]. The frequency of these black laminae reduced as they progressed upwards until they entirely vanished at a depth of around 370 cm. Hence, we ascribe the section between 1 to 750CE (~370cm to 232cm) as very likely annually laminated.

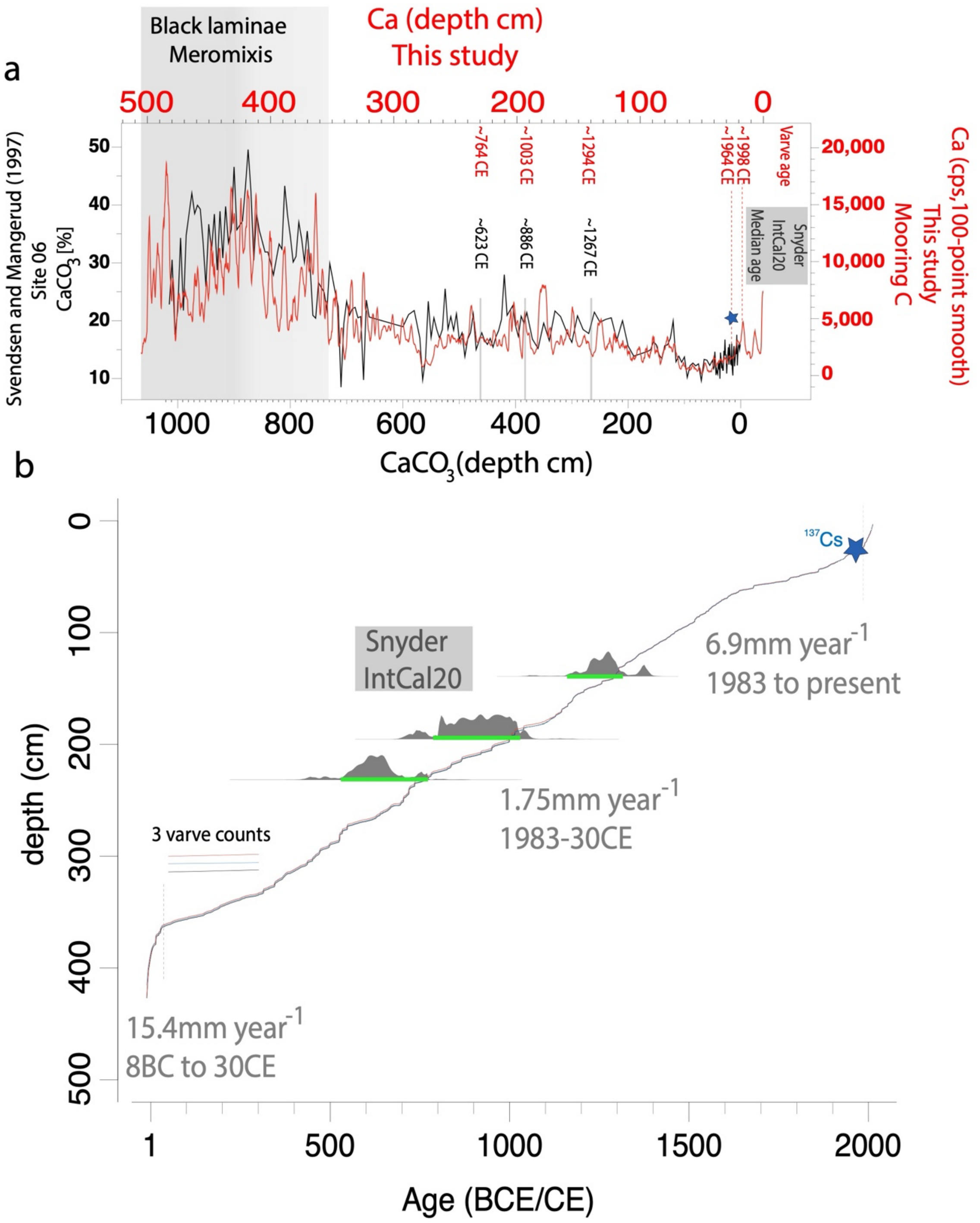

Figure S17. **a** comparison between µ-XRF Ca (filtered using a 100-point running mean and depicted in red) and the percentage of carbonates ($CaCO_3$ percent) from Svendsen and Mangerud[6]. The median calibrated radiocarbon dates are displayed, accompanied by varve ages indicated in red (representing the mean age of the three varve counts). **b** Age model constructed based on the three counts. The presence of the ^{137}Cs peak in our record is denoted by a blue star, identified through stratigraphic correlation with a recent core from Linnévatnet[4]. Additionally, the three calibrated ^{14}C ages from Snyder et al.[5] are depicted with 90 percent probability in green.

Supplementary note 2

Pairing the intervalometer findings with meteorological data enables the temporal alignment of sedimentary occurrences. The intervalometer, deployed on 7/25/2016 and retrieved on 7/26/2017, monitored sedimentation at mooring C in 30-minute intervals. Within the intervalometer data, a consistent voltage recording at 2.45V indicated an overflow event on 10/15/2016, or 15 October 2016 (Fig. S17). Variations in voltage within the intervalometer corresponded to sediment depth, and leveraging this relationship allows for the dating of sedimentary events within the intervalometer receiving tube.

Interval 1 encompasses all sedimentation recorded from the deployment on 7/25/2016 until 10/14/2016, representing the initial ~64mm of sediment within the intervalometer receiving tube. Interval 2 shows an extensive sedimentary event of 81+ mm that occurred within a single day, overwhelming the intervalometer on 10/15/2016. This event accounted for a minimum of 56% of the total sediment received in the intervalometer. It can be seen that the unprecedented rain event from October 15, 2016 was recorded by both an increase of grain-size and Ca (Fig. S18; lower panel). In contrast, no important increase in grain-size is observed during daily rainfall events that were of smaller magnitude (10-20mm) in interval 1 (I1).

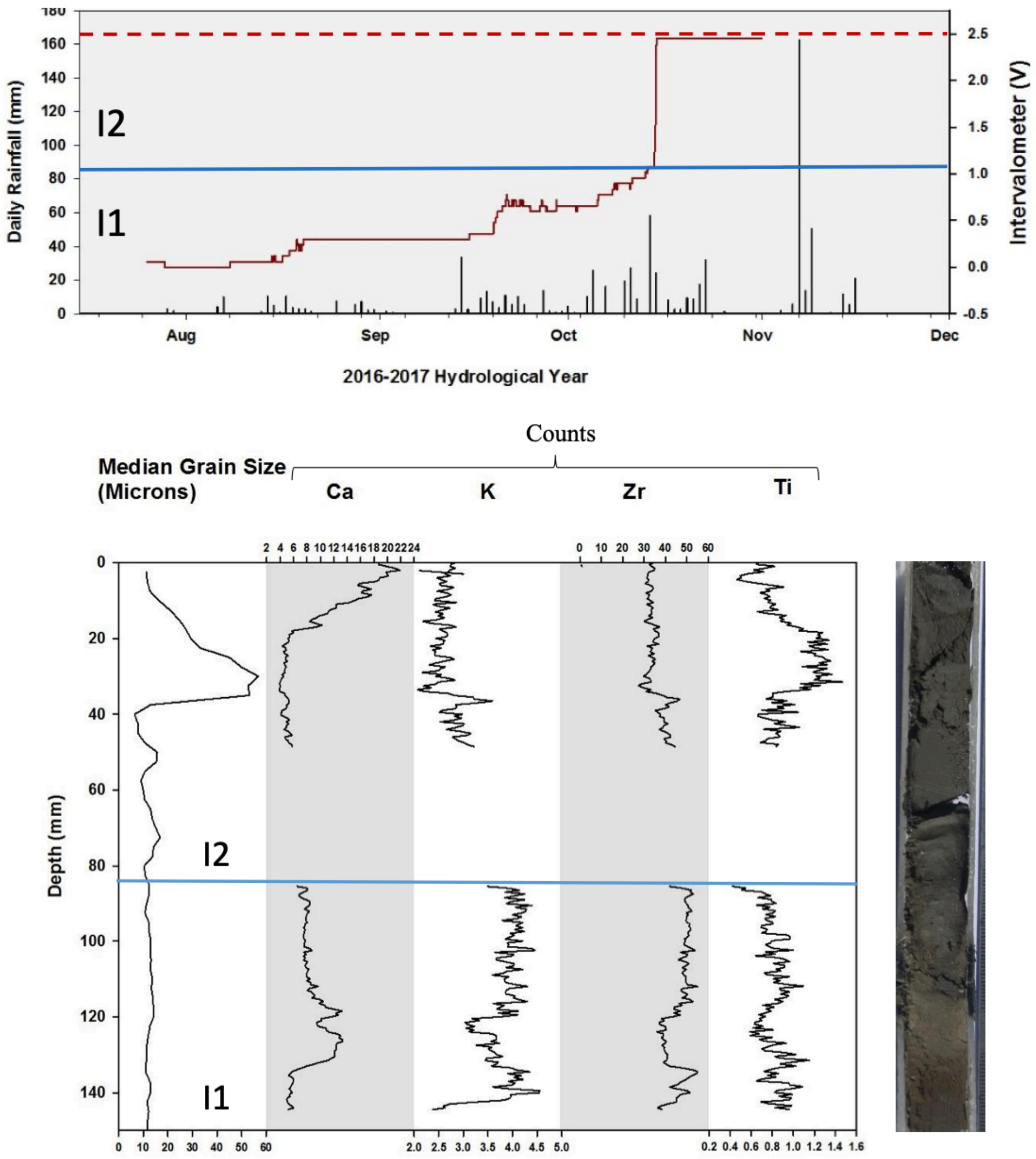

Figure S18. Upper panel: Graph displaying the daily rainfall (depicted in black) and intervalometer data (shown in red) during the autumn of 2016. Within this timeframe, Intervals 1, 2, and 3 represent distinct sedimentary events. Daily rainfall (black) and intervalometer data (red) for the fall of 2016. Intervals 1 and 2 are sedimentary events. Lower panel: A composite log detailing the intervalometer, which experienced overflow on October 15, 2016. This intervalometer is situated adjacent to mooring C, with specific annotations for Intervals 1 and 2. Figures modified from McGinn (2018)[9].

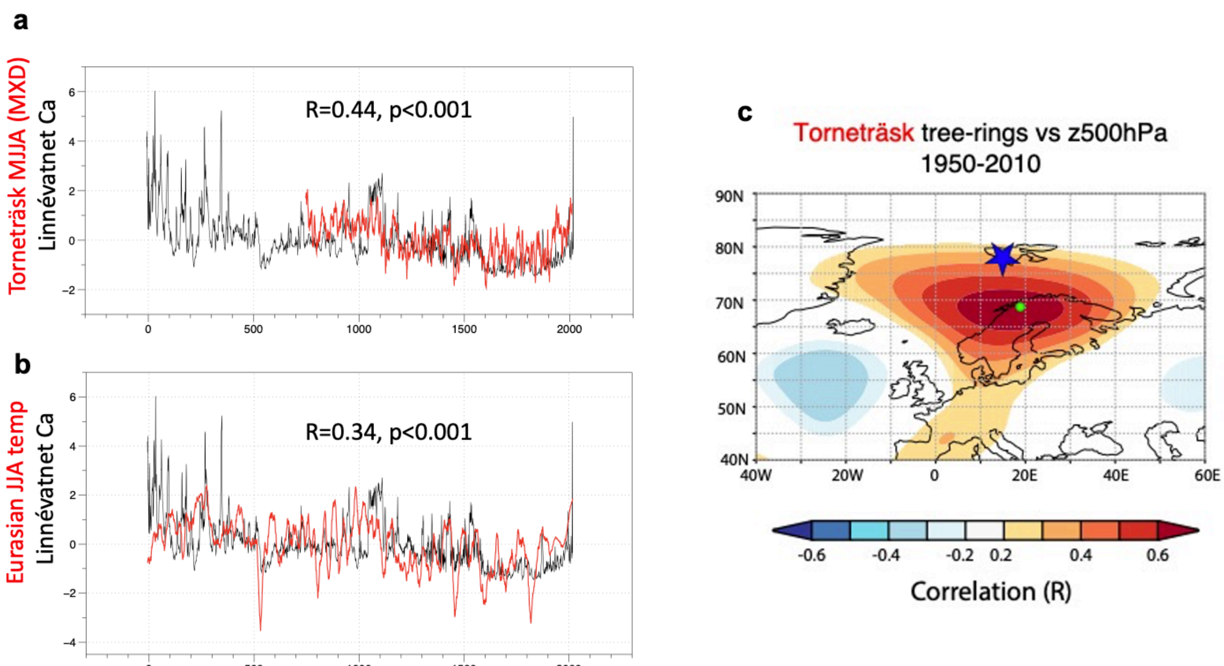

Figure S19. Same as Fig. 3, but using MXD JJA from Tornesträsk tree-rings (**a**) and **b**, same as **a**, but for tree-rings located in Eurasia (EA) with the period 2017-1901 corresponds to instrumental gridded data [10]. In **a** and **b**, tree-ring time series are filtered by a 5-year running mean (**a**) and an 11-year running mean (**b**) to improve visibility, respectively. **c,** Spatial correlation between Tornesträsk tree-rings and atmospheric pressure at z500hPa. The blue star is the location of Linnévatnet, 44 km south of Longyearbyen.

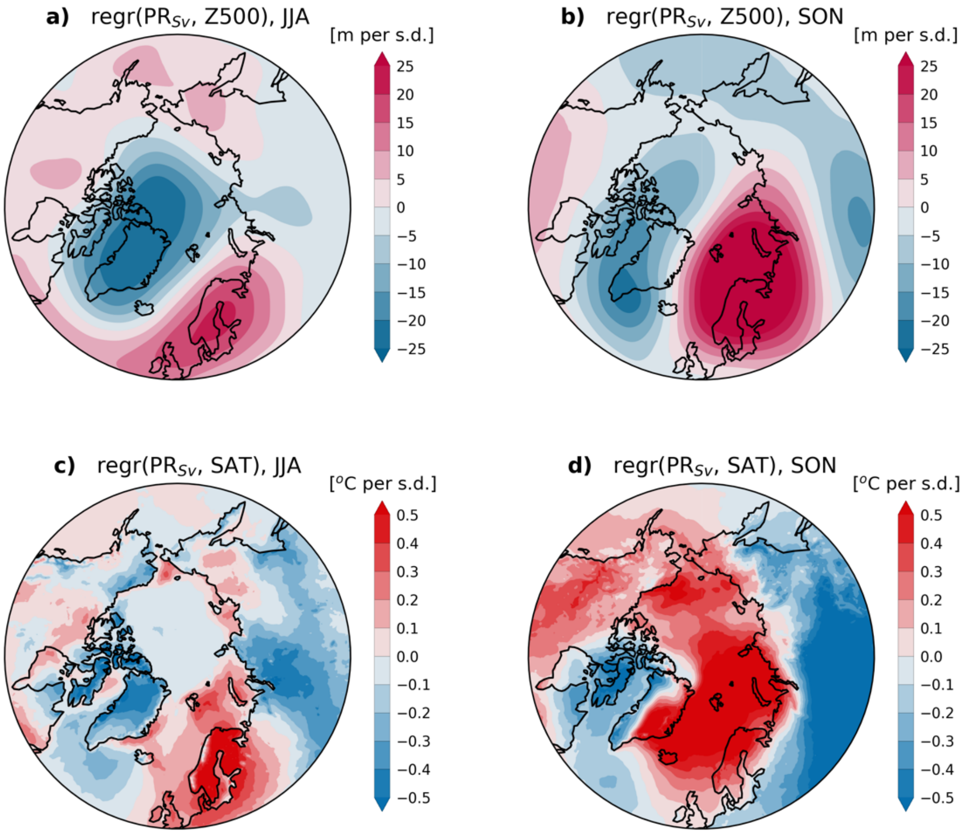

Figure S20. The relationships between Svalbard precipitation and Z500 and SAT for JJA and SON separately. Same as Figs. 1b and 1d in the main text.

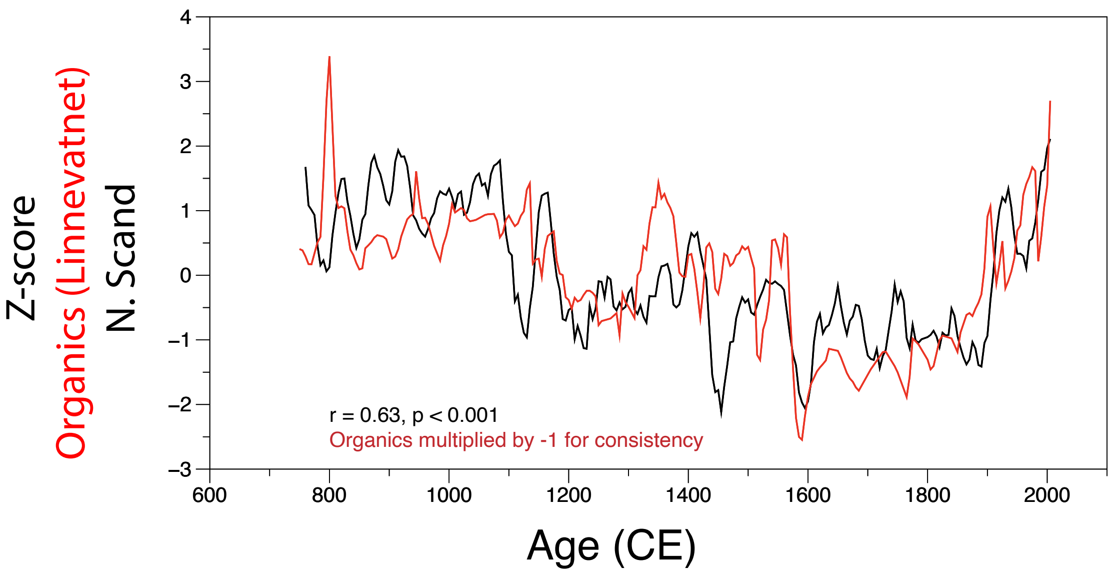

Figure S21. Organic concentration at Linnévatnet[4] and N-Trend Scand from tree-rings[11]. Data are interpolated at the lowest temporal resolution (organics), i.e., 8 years. Organic data multiplied by -1 for consistency.

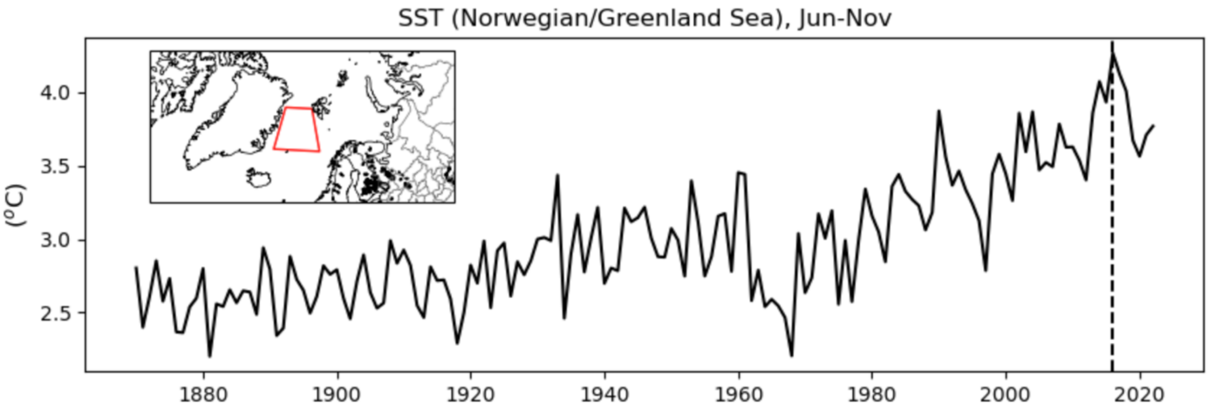

Figure S22. Jun-Nov mean SST for the period 1871-2021 based on HadISST[12] gridded product at 1˚x1˚ resolution. Note that the ERSST v5 yields similar results.

Supplementary References

1. Dobler, A., Førland, E. & Isaksen, K. Present and future heavy rainfall statistics for Svalbard–Background-report for Climate in Svalbard 2100. *Norwegian Center for Climate Services (NCCS) report* (2019).
2. Kalnay, E. *et al.* The NCEP/NCAR 40-year reanalysis project. *Bulletin of the American meteorological Society* **77**, 437-471 (1996).
3. Williams, G. Paleoenviromental Reconstruction from the Sediment Record of the Varved Proglacial Linnévatnet, Svalbard, Norwegian High Arctic. (2017).
4. Lapointe, F. *et al.* Multi-proxy evidence of unprecedented hydroclimatic change in a high Arctic proglacial lake: Linnévatnet, Svalbard. *Arctic, Antarctic, and Alpine Research* **55**, 2223403, doi:10.1080/15230430.2023.2223403 (2023).
5. Snyder, J., Miller, G., Werner, A., Jull, A. & Stafford Jr, T. AMS-radiocarbon dating of organic-poor lake sediment, an example from Linnévatnet, Spitsbergen, Svalbard. *The Holocene* **4**, 413-421 (1994).
6. Svendsen, J. I. & Mangerud, J. Holocene glacial and climatic variations on Spitsbergen, Svalbard. *The Holocene* **7**, 45-57 (1997).
7. Ramsey, C. B. Deposition models for chronological records. *Quaternary Science Reviews* **27**, 42-60 (2008).
8. Reimer, P. J. *et al.* The IntCal20 Northern Hemisphere radiocarbon age calibration curve (0–55 cal kBP). *Radiocarbon* **62**, 725-757 (2020).
9. McGinn, G. H. Sediment trap analysis in high-arctic lake Linnévatnet indicates a recent shift in the annual hydrological regime. (2018).
10. Büntgen, U. *et al.* Prominent role of volcanism in Common Era climate variability and human history. *Dendrochronologia* **64**, 125757 (2020).
11. Anchukaitis, K. J. *et al.* Last millennium Northern Hemisphere summer temperatures from tree rings: Part II, spatially resolved reconstructions. *Quaternary Science Reviews* **163**, 1-22 (2017).
12. Rayner, N. *et al.* Global analyses of sea surface temperature, sea ice, and night marine air temperature since the late nineteenth century. *Journal of Geophysical Research: Atmospheres* **108** (2003).
